# Axillary ultrasound for predicting response to neoadjuvant treatment in breast cancer patients—a single institution experience

**DOI:** 10.1186/s12957-023-03174-8

**Published:** 2023-09-16

**Authors:** Nina Pislar, Gorana Gasljevic, Maja Marolt Music, Simona Borstnar, Janez Zgajnar, Andraz Perhavec

**Affiliations:** 1 Department of Surgical Oncology, Institute of Oncology Ljubljana, Zaloska cesta 2, 1000 Ljubljana, Slovenia; 2 Medical Faculty, University of Ljubljana, Ljubljana, Slovenia; 3 Department of Pathology, Institute of Oncology Ljubljana, Ljubljana, Slovenia; 4 Department of Radiology, Institute of Oncology Ljubljana, Ljubljana, Slovenia; 5 Department of Medical Oncology, Institute of Oncology Ljubljana, Ljubljana, Slovenia

**Keywords:** Breast cancer, Neoadjuvant therapy, Axillary ultrasound, Axillary staging, Sentinel lymph node biopsy

## Abstract

**Background:**

In node-positive breast cancer patients at diagnosis (cN +) that render node-negative after neoadjuvant systemic treatment (NAST), axillary lymph node dissection (ALND) can be avoided in selected cases. Axillary ultrasound (AUS) is most often used for re-staging after NAST. We aimed to determine sensitivity, specificity, positive predictive value (PPV), and negative predictive value (NPV) of AUS after NAST for predicting nodal response at the Institute of Oncology, Ljubljana.

**Methods:**

Biopsy-confirmed cN + patients consecutively diagnosed at our institution between 2008 and 2021, who received NAST, followed by surgery were identified retrospectively. Only patients that underwent AUS after NAST were included. AUS results were compared to definite nodal histopathology results. We calculated sensitivity, specificity, PPV and NPV of AUS. We also calculated the proportion of patients with false-positive AUS that results in surgical overtreatment (unnecessary ALND).

**Results:**

We identified 437 cN + patients. In 244 (55.8%) AUS after NAST was performed. Among those, 42/244 (17.2%) were triple negative (TN), 78/244 (32.0%) Her-2 positive (Her-2 +), and 124/244 (50,8%) luminal Her-2 negative cancers. AUS was negative in 179/244 (73.4%), suspicious/positive in 65/244 (26.6%) (11/42 (26.2%) TN, 19/78 (24.4%) Her-2 + , and 35/124 (28.2%) luminal Her-2 negative cancers). On definite histopathology, nodal complete response (pCR) was observed in 89/244 (36.5%) (19/42 (45.2%) TN, 55/78 (70.5%) Her-2 + , and 15/124 (12.1%) luminal Her-2 negative cancers). Among patients with suspicious/positive AUS, pCR was observed in 20/65 (30.8%) (6/11 (54.5%) TN, 13/19 (68.4%) Her-2 + and 1/35 (2.9%) luminal Her-2 negative cancers). Sensitivity was 29.0%, specificity 77,5%, PPV 69.2%, NPV 38.5%. Specificity and PPV in TN was 68.4% and 45.4%, in Her-2 + 76.4% and 31.6%, in luminal Her-2 negative 93,3% and 97,1%, respectively.

**Conclusion:**

In approximately half of the patients, AUS falsely predicts nodal response after NAST and may lead to overtreatment in 30% of the cases (ALND). However, AUS has to be interpreted in context with tumor subtype. In luminal Her-2 negative cancers, it has a high PPV and is therefore useful.

## Background

In breast cancer patients with initially node-positive disease (cN +) treated with neoadjuvant systemic treatment (NAST), re-staging is performed to assess nodal response after NAST [1]. Axillary surgery is planned accordingly; if residual nodal disease is still clinically present (ycN +), axillary lymph node dissection (ALND) is performed, while in case of complete clinical response (ycN0), sentinel lymph node biopsy (SLNB) with removal of at least three nodes or targeted axillary dissection (TAD) is an established method with acceptably low false-negative rates [2–4]. Performing SLNB only in ycN0 patients is oncologically safe with locoregional recurrence rates below 2% after 10 years of follow-up [5].

Pathologic complete response (pCR) in the axilla is expected in up to 70% of cases, but not all are successfully spared ALND [6]. In addition to axillary palpation, axillary ultrasound (AUS) is recommended for re-staging [7]. According to the literature, sensitivity and specificity of AUS for predicting nodal response to NAST are different across studies and institutions (sensitivity 37–100%, specificity 69–92%) [8]. With an accuracy of up to 70%, AUS potentially leads to an incorrect surgical strategy in approximately one third of cases [9]. The criteria for suspicious/positive lymph nodes in the post-neoadjuvant setting also differ between institutions reported in the literature. Therefore, the institution’s own data are important for surgical planning.

The primary aim of our study was to determine the sensitivity, specificity, positive predictive value (PPV), and negative predictive value (NPV) of AUS after NAST for predicting nodal response at the Institute of Oncology Ljubljana. Secondary aim was to determine the proportion of patients with false-positive AUS leading to overtreatment (unnecessary ALND).

## Methods

We retrospectively identified female patients that were consecutively diagnosed with node-positive breast cancer at the Institute of Oncology Ljubljana, Slovenia, from January 2008 to December 2021, who received NAST that was followed by surgery. Positive nodal status at diagnosis (cN +) was determined as a positive fine-needle aspiration biopsy (FNAB) result. cN + patients at diagnosis in whom AUS was performed after NAST before surgery were eligible for analysis. AUS was performed by several different radiologists, all of whom had experience in breast pathology. Lymph nodes were classified as negative or suspicious/positive. The criteria for pathological lymph nodes on AUS were as previously described: longitudinal-transverse ratio less than 1.5, loss of fatty hilus, or cortical thickness greater than 3 mm [10].

AUS results were compared with final histopathology results (gold standard). We calculated sensitivity, specificity, PPV, and NPV of AUS after NAST for predicting nodal response. Among patients with suspicious/positive AUS results, we calculated the proportion of patients who achieved a pathologic complete response (pCR). These are the patients who may undergo unnecessary ALND if surgery is planned based on the AUS results.

The study was approved by the institutional review board and ethics committee. Informed consent was not required because of the retrospective nature of the study.

## Results

**Fig. 1:**
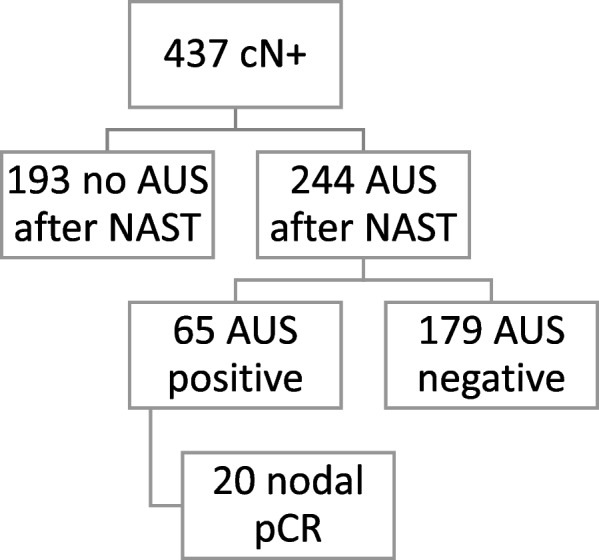
Patients*’* flow (AUS—axillary ultrasound, cN + —clinically positive lymph nodes at presentation, NAST—neoadjuvant systemic treatment, pCR—pathologic complete response)

We identified 437 cN + female patients treated with NAST, followed by surgery. Patient flow is visualized in Fig. 1.

**Fig. 2:**
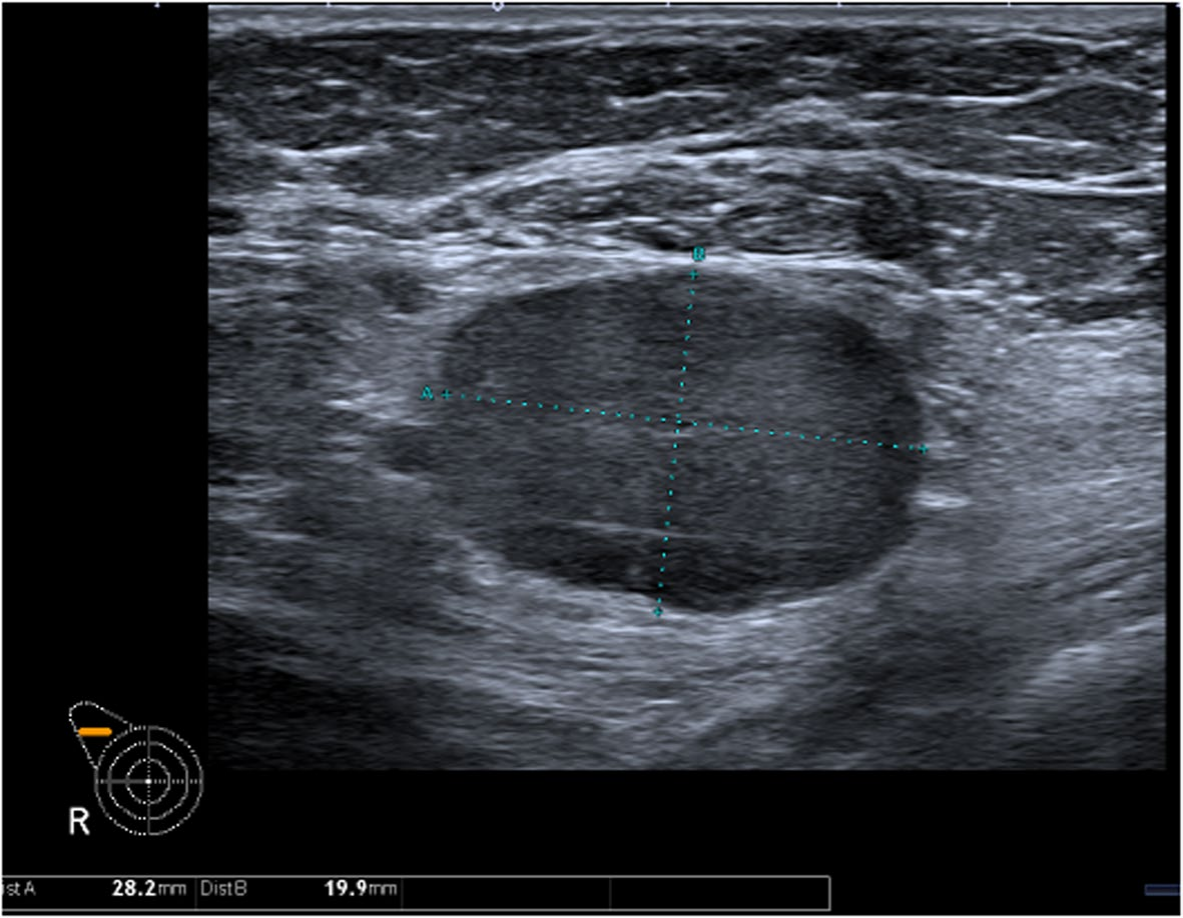
Pathological axillary lymph node on ultrasound before neoadjuvant systemic treatment measuring 28 × 20 mm

**Fig. 3:**
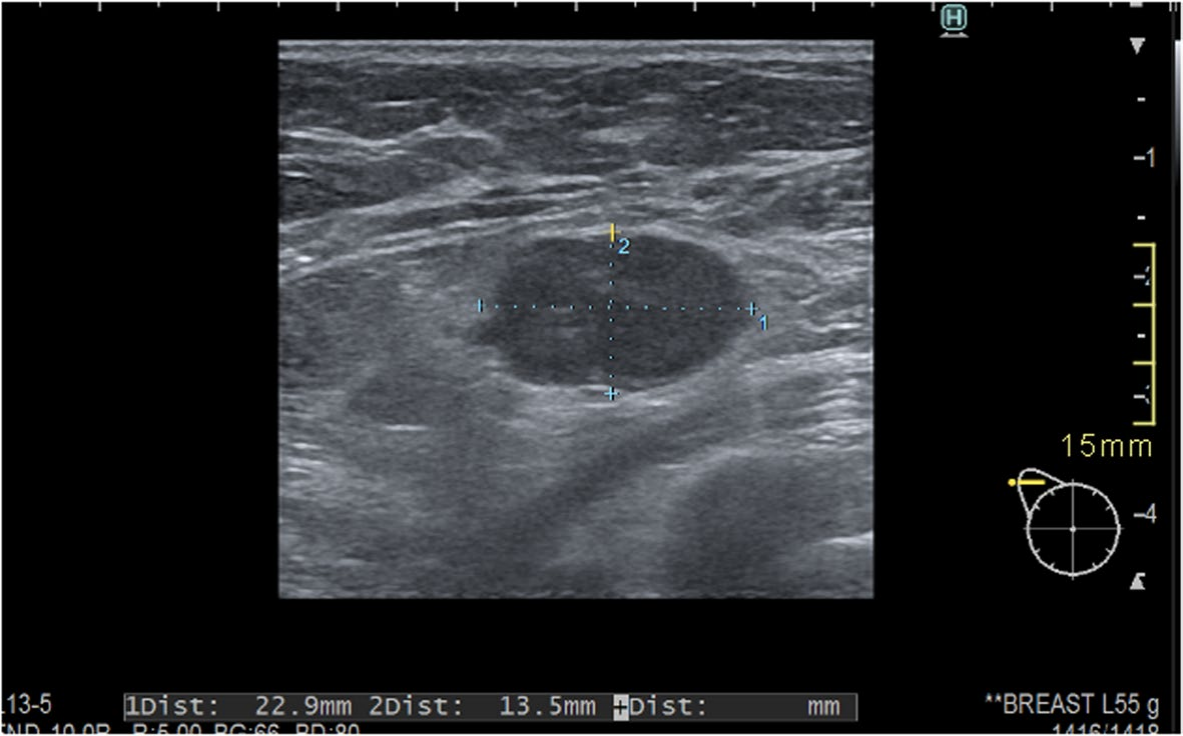
Persistent pathologic axillary lymph node on ultrasound after neoadjuvant systemic treatment measuring 23 × 13.5 mm

In 244 (55.8%) AUS after NAST was performed. Patients’ characteristics are presented in Table 1. Among 244, AUS was negative in 179/244 (73.4%) and suspicious/positive in 65/244 (26.6%). Figures 2 and 3 represent AUS images of a suspicious/pathological axillary lymph node at diagnosis and post-NAST, respectively.

**Table 1:** Patients’ characteristics

	*N* = 244
Age (median, range)	48 (27–78)
Subtype	
*Luminal*	*124 (50.8%)*
*Her-2* +	*78 (32.0%)*
*TN*	*42 (17.2%)*
Histology	
*Invasive ductal carcinoma*	*234 (95.9%)*
*Invasive lobular carcinoma*	*7 (2.9%)*
*Other*	*3 (1.2%)*
Tumor size at presentation^a^	31 (4–100)
Tumor size after NAST^a^	10 (0–85)

*Her-2* human epidermal growth factor receptor 2, *NAST* neoadjuvant systemic treatment, *TN* triple negative

^a^Median tumor size on ultrasound or magnetic resonance imaging in milimeters with range

On final histopathology nodal pCR was achieved in 89/244 (36.5%) patients. In patients with suspicious/positive US nodal pCR was achieved in 20/65 (30.8%) (Table 2). In 91/244 (37.3%) patients, we performed primary ALND. In 153/244 (62.7%) patients that underwent SNB, we proceeded to ALND in 71/153 cases (during the same procedure in 29/153 (18.9%) cases (positive imprint cytology) and in 42/153 (27.4%) cases during a separate procedure).

**Table 2:** Patients by subtypes

	*N* = 244	%
Positive AUS	65	26.6
*Luminal*	*35*	*28.2*
*Her-2* +	*19*	*24.4*
*TN*	*11*	*26.2*
pCR	89	36.5
*Luminal*	*15*	*12.1*
*Her-2* +	*55*	*70.5*
*TN*	*19*	*45.2*
pCR in positive AUS	20	30.8
*Luminal*	*1*	*2.9*
*Her-2* +	*13*	*68.4*
*TN*	*6*	*54.5*

*AUS* axillary ultrasound, *Her-2* human epidermal growth factor receptor 2, *pCR* pathologic complete response, *TN* triple negative

AUS correctly predicted nodal response to NAST in 46.7% of patients (accuracy). Predictive parameters of AUS are shown in Table 3.

**Table 3:** Predictive parameters of axillary ultrasound

	Sensitivity	Specificity	PPV	NPV
All	29.0%	77.5%	69.2%	38.5%
Luminal	32.4%	93.3%	97.1%	16.9%
Her-2 +	27.3%	77.4%	33.3%	71.9%
TN	21.7%	68.4%	45.4%	41.9%

*Her-2* human epidermal growth factor receptor 2, *NPV* negative predictive value, *PPV* positive predictive value, *TN* triple negative

## Discussion

AUS is a noninvasive and readily available imaging tool that guides surgical planning in upfront surgery. However, it is only moderately sensitive and specific for differentiating between high and low tumor burden in the axilla, and leads to overtreatment in more than 50% of cases when Z011 criteria are applied [11]. We have previously shown that performing pre-operative AUS is not necessary in non-palpable screen-detected cancers treated with upfront surgery, as it may lead to overtreatment [12].

For cN + patients that undergo NAST, nodal pCR is expected in 18–60% of patients [13]. The goal is to avoid unnecessary ALND in these patients. If nodal pCR is expected after NAST, it is reasonable to perform SNB with removal of at least 3 nodes [14–16]. However, if the residual nodal burden is high, the FNR is above the acceptable 10% [17]. Thus, in cN + patients, appropriate surgical planning with re-staging after NAST is important.

In our series, PPV and NPV for AUS were 69% and 38.5%, respectively. In comparison to 715 cN + patients from SENTINA trial (PPV 77%, NPV 50%), AUS performed worse in our series. Their inclusion criteria for positive nodal status did not include positive FNAB result at presentation, which can explain a higher proportion of negative nodes on definite histology (pCR) than in our series (36% vs. 46%) and hence better NPV [18]. AUS at our institution had a sensitivity and specificity for predicting pCR of 29% and 77.5%, respectively, while in other series with comparable pCR rates to ours, sensitivity and specificity were 59–70% and 58–79%, respectively [9, 19–21]. PPVs were more variable in other series (65–83%), which can be explained by radiologists’ variability [8].

US-guided FNAB after NAST is not routinely performed at our institution. It is an invasive procedure that adds time and cost. It is also limited by non-diagnostic results. However, US-guided FNAB has a low false-positive rate of only 0–8%, suggesting patients with a positive FNAB after NAST can safely proceed to ALND [22, 23]. According to our data, if AUS alone is used to guide surgical decision making, it leads to overtreatment in the axilla in almost one third of cases.

In our study, the criteria for suspicious/positive lymph nodes were inconsistently reported, which is consistent with the fact that AUS results are highly operator dependent. Since the criteria for suspicious/positive nodes we used apply to upfront surgery, sensitivity of AUS would most likely improve with adjusted criteria after NAST, such as taking into account also eccentric cortical thickening, indistinct margins, perinodal edema [20].

Interestingly, in our study AUS had a very high predictive value for residual nodal disease in luminal, Her-2 negative cancers (PPV 97%), which is in accordance with the study of Di Micco et al. [9]. This is not surprising, since only 12% of luminal, Her-2 negative cancers achieved pCR in our series. On the other hand, NPV was the highest for Her-2 positive subgroup of patients (NPV 72%), which is also expected due to pCR rates of more than 60% in these patients [6, 13]. Di Micco et al. and Maeshima et al. both reported AUS performance was different across different tumor subtypes [9, 21].

While ultrasound is invaluable in detecting axillary involvement at diagnosis, our data showed that it is not an optimal re-staging method after NAST. However, among commonly used imaging modalities in the neoadjuvant setting, ultrasound does have the best predictive power, is readily available, non-invasive, and remains the imaging method of choice for re-evaluating the axilla in the neoadjuvant setting. Alternatively, predictive value of magnetic resonance imaging (MRI) or positron emission tomography/computer tomography (PET) has been shown to be better than AUS in some smaller series [20, 24]. Nevertheless, routine use of PET for re-staging at our institution is limited by the availability of the method for a questionable added benefit.

However, according to our results ultrasound findings must be interpreted in the context of the clinicopathological characteristics of the tumor that predict response to neoadjuvant treatment. The probability of achieving axillary pCR varies among tumor subtypes (highest in Her-2-positive subtype, lowest in luminal Her-2-negative subtype) [13]. Axillary pCR rates for the TN subtype are expected to improve further with the use of chemo-immunotherapy (which was not yet used during our study period).

If other clinicopathological characteristics in addition to tumor subtype were included in the prediction model (tumor size, receptor status, tumor-infiltrating lymphocytes, lymphovascular invasion, etc.) the probability of axillary pCR could be predicted with higher accuracy [25]. When this is combined with AUS results, axillary surgical staging can be optimized. An ideal model for predicting axillary status after NAST could lead to avoiding axillary surgery altogether in patients with axillary pCR on the one hand and performing axillary dissection directly in patients with residual axillary metastases on the other hand. Several models have already been developed to predict nodal response after NAST that include clinicopathologic characteristics with or without AUS [26–29]. However, AUC values are less than 0.8 and the models often lack external validation.

Study limitations include the retrospective analysis and the long period we analyzed. There was heterogeneity in NAST treatment protocols and eligibility criteria for receiving NAST (selection bias), which changed over the years. The criteria for suspicious/pathologic lymph nodes are also an important limitation. The strengths of the study area relatively large number of patients from a single high-volume breast center and no missing data despite the retrospective nature of the study.

Our study represents a “real-life” analysis of how a certain imaging modality could lead to overtreatment which is a common phenomenon in oncology. Because of retrospective nature and long period, high number of radiologists participating in the study and operator dependent nature of AUS, the study was not designed to universally change the preoperative practice in breast cancer patients after NAST. Instead, it is a retrospective review of how often AUS led to unnecessary ALND in a single institution with its own specifics. Based on high proportion of unnecessary ALND as a result of false-positive AUS in our series, other institutions are encouraged to do similar analysis and act accordingly to their own results.

## Conclusion

In approximately half of the cases, AUS incorrectly predicted nodal response to NAST. This potentially leads to overtreatment (unnecessary ALND) in almost 30% of cases. However, we can conclude that according to our results in luminal Her-2 negative tumors, AUS has a very high PPV and it is reasonable for these patients to proceed directly to ALND if the AUS result is positive. On the other hand, in Her-2-positive tumors AUS has high NPV and if AUS shows axillary disease elimination, it is reasonable to consider SLNB only, even if less than 3 lymph nodes are retrieved. This is a single center experience and other institutions are encouraged to do their own analysis.

## Data Availability

The datasets generated during and/or analysed during the current study are available from the corresponding author on reasonable request.
